# Digits and Fin Rays Share Common Developmental Histories

**DOI:** 10.1038/nature19322

**Published:** 2016-08-17

**Authors:** Tetsuya Nakamura, Andrew R. Gehrke, Justin Lemberg, Julie Szymaszek, Neil H. Shubin

**Affiliations:** Department of Organismal Biology and Anatomy, University of Chicago, Chicago IL, 60637, USA

Understanding the transformation of fish fins into tetrapod limbs is a fundamental problem in biology^1^. The search for antecedents of tetrapod digits in fish has remained controversial because the distal skeletons of limbs and fins differ structurally, developmentally, and histologically^2,3^. Moreover, comparisons of fins with limbs have been limited by a relative paucity of data on the cellular and molecular processes underlying the development of the fin skeleton. Here, we provide the first functional analysis, using CRISPR/Cas9 and fate mapping, of 5′ *hox* genes and enhancers in zebrafish that are indispensable for the development of the wrists and digits of tetrapods^4,5^. We show that the fates of cells marked by the activity of an autopodial *hoxa13* enhancer exclusively form elements of the fin fold, including the osteoblasts of the dermal rays. In *hox13* knockout fish, we find that a dramatic reduction and loss of fin rays is associated with an increased number of endochondral distal radials. These discoveries reveal a deep cellular and genetic connection between fin rays of fish and the digits of tetrapods and suggest a mechanism of digit origins via the transition of distal cellular fates.

The origin of tetrapod limbs involved profound changes to the distal skeleton of fins. Fin skeletons are composed mostly of fin rays^6^, whereas digits are the major anatomical and functional components of the distal limb skeleton. One of the central shifts during the origin of limbs in the Devonian period involved the reduction of fin rays coincident with an expansion of the distal endochondral bones of the appendage^2,7^. Because the distal skeletons of fins and limbs are composed of different types of bone tissue (dermal and endochondral, respectively) it remains unclear how the terminal ends of fish and tetrapod appendages are related and, consequently, how digits arose developmentally. While an understanding of ectodermal signaling centers in fin buds and fin folds has advanced in recent years^8–11^, that of the cells that actually form the skeletal patterns has remained elusive.

*Hox* genes, namely those of the *HoxA* and *HoxD* clusters, have figured prominently in discussions of limb development and origins^3,12–14^. An “early” and “late” phase of *HoxD* and *HoxA* transcription play a role in specifying the proximal (arm and forearm) and distal (autopod) segments, respectively^15^. Both fate map assays and knockout phenotypes in mouse limbs reveal an essential role for *Hox13* paralogues in the formation of the autopod^4,5^. Mice engineered to lack *Hoxa13* and *Hoxd13* in limbs lack the wrists and digits exclusively^4^. Moreover, the lineage of cells expressing *Hoxa13* reside exclusively in the autopod of adult mice^5^. Together, these lines of evidence reveal the extent to which 5′ *Hox* genes are involved in, and serve as markers for, the developmental pattern of wrist and digits. Unfortunately, since no such studies have yet been performed in fish, the means to find antecedents of autopodial development in fins has been lacking.

Analyses of 5′ *Hox* expression in phylogenetically diverse wild-type fish^16–19^ as well as experimental misexpression in teleosts reveal that 5′ *Hox* activity may be involved in patterning^20^, and defining the extent of, the distal chondrogenic region of fish fins^21^. Despite these advances, however, little is known about the contribution of different *hox* paralogues—individually and in combination— to the adult fin phenotype and the origin of cells that give rise to the distal fin skeleton. While previous investigations have shown that osteoblasts of the fin rays in the caudal fin of zebrafish are derived from either neural crest or from paraxial mesoderm, the source of osteoblasts in pectoral fin rays is currently unknown^22–24^. Consequently, it remains an open question where the cellular and genetic markers of the autopod of the tetrapod limb reside in fish fins.

In order to bridge these gaps in knowledge, we followed the fates of cells marked by early and late phase *hox* enhancers to adult stages in pectoral fins. In addition, we engineered zebrafish that completely lack each individual *hox13* gene, and bred stable lines with multiple gene knockout combinations of *hox* paralogues. The power of these experiments is twofold: 1) they represent the first functional analyses of *hox* activity in fins, and 2) invite a direct developmental comparison to experiments performed in tetrapod limbs.

We performed *in situ* hybridization of *hox13 a* and *d* paralog group genes from 48-120 hours post fertilization (hpf) in zebrafish to determine if active *hox* expression has a role in the development of the pectoral fin fold. *Hox13 a* genes in zebrafish are expressed in the distal fin mesenchyme at 48 hpf and weakly in the proximal portion of the pectoral fin fold from 72-96 hpf, indicating that *hox13 a* genes are not actively expressed in the developing fold (Fig. 1a, b)^18^. *Hoxd13a* is expressed in the posterior half of the fin, but it becomes weak after 96 hpf (Fig. 1c). *Hox* expression is entirely absent in 10 days post fertilization (dpf) fins (Extended Data Fig. 1). As *hox13* genes do not appear to have a main role in zebrafish fin fold development past 72-96 hpf, we sought to determine what structures *hox* positive cells populate in the developing and adult fold.

To follow the fates of cells that experience early phase activity in the zebrafish fin, we modified our previously reported transgenesis vector^21^ to express *Cre*-recombinase driven by the zebrafish early phase enhancer CNS65^25^. This enhancer activates expression throughout the endochondral disk of pectoral fins from 31 to ∼38 hpf (Fig. 2a, Extended Data Fig. 1). Stable lines expressing CNS65x3-Cre were crossed to the lineage-tracing zebrafish line Tg(*ubi*:*Switch*) fish, where cells are permanently labeled with mCherry^26^. At 6 dpf mesenchymal cells that received expression driven by CNS65 at 38 hpf make up the entire endochondral disk of the pectoral fin (Fig. 2b). Surprisingly, we also found mCherry positive cells in the fin fold at 6 dpf and extensively at 20 dpf (Fig. 2b). These cells contained filamentous protrusions extending distally as well as nuclei positioned at the posterior side, both of which suggest that the cells are migrating distally out of the endochondral disk (Fig. 2b).

To determine the fate of late phase cellular activity, we employed the same fate mapping strategy but used a late phase *hoxa* enhancer (e16) from the spotted gar genome^21^. We chose a *hoxa* enhancer because lineage-tracing data in mouse has shown that late phase *Hoxa13* cells in the limb make up the osteoblasts of the wrist and digits exclusively, making it a *bona fide* marker of the autopod^5^. In addition, gar e16 (which has no sequence conservation in zebrafish) drives expression throughout the autopod in transgenic mice in a pattern that mimics the endogenous murine enhancer and *Hoxa13* expression^21,27^. In transgenic zebrafish, gar e16 is active in the distal portion of the endochondral disk of the pectoral fin at 48 hpf, and ceases activity after approximately 55 hpf (Fig. 2a, Extended Data Fig. 1). When crossed to Tg(*ubi*:*Switch*), at 6 dpf we detected the majority of mCherry positive cells in the developing fin fold with a small number of cells lining the distal edge of the endochondral disk (Fig. 2c). At 20 dpf, the fin fold contains nearly all of the mCherry positive cells that have formed tube-like cells that are appear to be developing actinotrichia (Fig. 2c). In adult fish (90 dpf), late phase cells are restricted to the adult structures of the fin fold, where they compose osteoblasts that make up the fin rays, among other tissues (Fig. 2d). As the e16 enhancer is only active in the distal endochondral disk at 48 hpf, and the labeled cells end up in the fin rays of the adult, late phase *hox*-positive cells are likely migrating from the endochondral portion of the fin into the fin fold, a hypothesis supported by extensive filopodia in mCherry positive cells projecting in the direction of the distal edge of the fin (Fig. 2c).

To explore the function of *hox13* genes, we inactivated individual *hox13* genes from the zebrafish genome by CRISPR/Cas9 and, in addition, made combinatorial deletions through genetic crosses of stable lines (Extended Data Fig. 2, Extended Data Table 2-4). Homozygous null embryos for individual *hox13* gene exhibited embryonic pectoral fins that were comparable in size with wild-type at 72 hpf (Extended Data Fig. 3). The shape and size of the fin fold and endochondral disk were also assayed by *in situ* hybridization for *and1* and *shha* genes, which serve as markers for the developing fin fold and endochondral disk, respectively (Extended Data Fig. 3)^28^. In adult fins (∼120 dpf), we observed no detectable difference in the length of fin rays of *hoxd13a*^-/-^ mutants when compared to wild-type (Fig. 3d, Extended Data Fig. 5). However, both *hoxa13a ^-/-^* and *hoxa13b ^-/-^* single mutant fish retained fin rays that were shorter than wild-type, suggesting a role for *hoxa13* genes in fin ray development (Fig. 3g, j, Extended Data Fig. 5). In order to determine the degree to which endochondral bones were affected, we utilized CT scanning technology for wild-type and mutant adult fish. Each single mutant, *hoxa13a*^-/-^,*a13b*^-/-^ or *d13a* ^-/-^, has four proximal radials and 6-8 distal radials with similar morphology to wild-type zebrafish (Fig. 3c, f, i, l, Extended Data Fig. 5). We crossed heterozygous mutants to obtain fish that completely lacked all *hoxa13* genes (*hoxa13a ^-/-^*,*a13b^-/-^*). The fin folds of *hoxa13a ^-/-^*,*a13b ^-/-^* embryos were ∼30% shorter at 72 and 96 hpf, whereas the number of cells in the endochondral disk was ∼10% greater than wild-type (Extended Data Fig. 4). Surprisingly, adult *hoxa13a ^-/-^*,*a13b ^-/-^* exhibited drastically reduced fin rays (Fig. 3m, Extended Data Fig. 5, Supplementary Information). In contrast to dermal reduction, the endochondral distal radials of double mutants were significantly increased to 10-13 in number, often stacked along the proximodistal axis (Fig. 3o, Extended Data Fig. 5, Supplementary Information). A similar pattern was seen in triple knockout fish (mosaic for hoxa13b and hoxd13a) (Fig. 3p-r, Extended Data Fig. 5), implying a role for late phase hox genes in patterning the proximal endochondral radials of fins that is unlike that of tetrapods (Fig. 3)

Despite being composed of different kinds of skeletal tissues, fin rays and digits share a common population of distal mesenchymal cells that experience late phase *Hox* expression driven by shared regulatory architectures and enhancer activities^21^. In addition, loss of 5′ *Hox* activity results in the deletion or reduction of both of these structures. Whereas phylogenetic evidence suggests that rays and digits are not homologous as morphological structures, the cells and regulatory processes in both the fin fold and the autopod share a deep homology that may be common for either bony fish or jawed vertebrates^19^.

Two major trends underlie the fin to limb transition--the elaboration of endochondral bones and the progressive loss of the extensive dermal fin skeleton^2,7,20^. In the combinatorial knockouts of *hox13* genes, which in tetrapods result in a loss of the autopod, distal endochondral radials were increased in number while fin rays were dramatically reduced. Since a common population of cells in the distal appendage is involved in the formation of rays and digits, the endochondral expansion in tetrapod origins may have occurred through the transition of distal cellular fates and differential allocation of cells from the fin fold to the fin bud^18^ (Fig. 4). The two major trends of skeletal evolution in the fin to limb transition may be linked at cellular and genetic levels.

## Methods

All zebrafish work was performed according to standard protocols approved by The University of Chicago (ACUP #72074).

### Whole mount *In situ* hybridization

*In situ* hybridization for the *hox13*, *Cre*, *and1* and *shha* genes were performed according to standard protocols^29^ after fixation in 4 % paraformaldehyde overnight at 4° C. Probes for *hox13* and *shha* were as previously described^18^. Primers to clone *Cre* and *and1* into vectors can be found in Extended Data Table 1 and 2. Specimens were visualized on a Leica M205FA microscope.

### Lineage tracing vector construction

In order to create a destination vector for lineage tracing, we first designed a random sequence of 298 bp that contained a SmaI sites to be used in downstream cloning. This sequence was ordered as a gBlocks fragment (IDT) and ligated into the pCR8/GW/TOPO TA cloning vector (Invitrogen). We then performed a Gateway LR reaction according to the manufacturers specifications between this entry vector and pXIG-cFos-GFP, which abolished an NcoI site present in the gateway cassette and introduced an SmaI site. We then removed the GFP gene with NcoI and BglII of the destination vector and ligated in *Cre* with (primers in Extended Data Table 1), using the “*pCR8GW-Cre*-pA-*FRT*-*kan*-*FRT*” (kind gift of Maximiliano L. Suster, Sars International Center for Marine Molecular Biology, University of Bergen, Bergen, Norway) as a template for *Cre* PCR and Platinum Taq DNA polymerase High Fidelity (Invitrogen). In order to add a late phase enhancer to this vector, we first ordered four identical oligos (IDT gBlocks) of the core e16 sequence from gar, each flanked by different restriction sites. Each oligo was then ligated into pCR8/GW/TOPO, and sequentially cloned via restriction sites into a single pCR8/GW/TOPO vector. This entry vector was used a template to PCR the final *Lo*-e16x4 sequence and ligate into the *Cre* destination vector using XhoI and SmaI, creating *Lo*-e16x4-Cre. The early phase enhancer *Dr*-CNS65x3 was cloned into the destination vector using the same strategy. Final vectors were confirmed by sequencing. A full list of sequences and primers used can be found in Extended Data Table 1.

### Establishment of lineage tracing lines

*AB zebrafish embryos were collected from natural spawning and injected according to the Tol2 system as described previously^21^. Transposase RNA was synthesized from the pCS2-zT2TP vector using the mMessage mMachine SP6 kit (Ambion)^21^. All injected embryos were raised to sexual maturity according to standard protocols. Adult F0 fish were outcrossed to wild-type *AB, and the total F1 clutch was lysed and DNA isolated at 24 hpf for genotyping (see Extended Data Table 1 for primers) to confirm germline transmission of *Cre* plasmids in the F0 founders. Multiple founders were identified and tested for the strongest and most consistent expression via antibody staining and *in situ* hybridization. One founder fish was identified as best, and all subsequent experiments were performed using these individual fish.

### Lineage tracing crossing and detection

Founder *Lo*-e16x4-Cre and *Dr*-CNS65x3-Cre fish were crossed to the Tg(*ubi*:*Switch*) line (kind gift of Leonard I. Zon, Stem Cell Program, Children's Hospital Boston, Boston, MA). Briefly, this line contains a construct where a constitutively active promoter (*ubiquitin*) drives expression of a loxP flanked GFP protein in all cells assayed of the fish. When *Cre* is introduced, the GFP gene is removed and the ubiquitin promoter is exposed to mCherry, thus permanently labeling the cell. We crossed our founder *Cre* fish to Tg(*ubi*:*Switch*) and fixed progeny at different time points to track cell fate. In order to detect mCherry signal, embryos or adults were fixed overnight in 4 % paraformaldehyde and subsequently processed for whole-mount antibody staining according to standard protocols^30^ using the following antibodies and dilutions: 1^st^ rabbit anti-mCherry/DsRed (Clontech #632496) at 1:250, 1^st^ mouse anti-zns-5 (Zebrafish International Resource Center, USA) at 1:200, 2^nd^ goat anti-rabbit Alexa Fluor 546 (Invitrogen #A11071) at 1:400, 2^nd^ goat anti-mouse Alexa 647 (Invitrogen #A21235) at 1:400. Stained zebrafish were mounted under a glass slide and visualized using an LSM 710 confocal microscope (Organismal Biology and Anatomy, the University of Chicago). Antibody stains on adult zebrafish (90 dpf) fins were imaged on a Leica SP5 II tandem scanner AOBS Laser Scanning Confocal (the University of Chicago Integrated Light Microscopy Core Facility).

### CRISPR/Cas9 design and synthesis

Two mutations were simultaneously introduced into the first exon of each *hox13* gene by CRISPR/Cas9 system as previously described in *Xenopus tropicalis*^31^. Briefly, two gRNAs that match the sequence of exon 1 of each *hox13* gene were designed by *ZiFiT* (http://zifit.partners.org/ZiFiT/). To synthesize gRNAs, forward and reverse oligonucleotides that are unique for individual target sequences were synthesized by Integrated DNA Technologies, Inc. (IDT). Each oligonucleotide sequence can be found in Extended Data Table 2. Subsequently, each forward and reverse oligonucleotide were hybridized, and double stranded products were individually amplified by PCR with primers that include a T7 RNA promoter sequence, followed by purification by NucleoSpin Gel and PCR Clean-up Kit (Macherey-Nagel). Each gRNA was synthesized from the purified PCR products by *in vitro* transcription with the MEGAscript T7 Transcription kit (Ambion). *Cas9* mRNA was synthesized by mMESSAGE mMACHINE SP6 Transcription Kit according to the manufacturer's instructions (Ambion).

### CRISPR/Cas9 injection and mutants selection

Two gRNAs targeting exon 1 of each *hox* gene were injected with *Cas9* mRNA into zebrafish eggs at the one cell stage. We injected ∼2 nL of the injection solution (5 μl solution containing 1000 ng of each gRNA and 500 ng *Cas9* diluted in nuclease free water) into the single cell of the embryo. Injected embryos were raised to adulthood, and at three months were genotyped by extracting DNA from tail clips. Briefly, zebrafish were anesthetized by Tricaine (0.004 %) and tips of the tail fin (2-3 mm square) were removed and placed in an eppendorf tube. The tissue was lysed in standard lysis buffer (10 mM Tris pH 8.2, 10 mM EDTA, 200 mM NaCl, 0.5 % SDS, 200 ug/mL proteinase K) and DNA recovered by ethanol precipitation. Approximately 800-1100 bp of exon 1 from each gene was amplified by PCR according to primers found in Extended Data Table 2. To determine if mutations were present, PCR products were subjected to T7E1 (T7 endonuclease1) assay as previously reported^32^. After identification of mutant fish by T7E1 assay, detailed analysis of mutation patterns were performed by sequencing at the Genomics Core at the University of Chicago.

### Establishment of *hox13* single and double mutant fish

Identified mutant fish were outcrossed to wild-type to select frameshift mutations from mosaic mutational patterns and establish single heterozygous lines. Obtained embryos were raised to adults (∼ three months), then analyzed by T7E1 assay and sequenced. Among a variety of mutational patterns, fish that have frameshift mutations were used for assays as single heterozygous fish. We obtained several independent heterozygous mutant lines for each *hox13* gene to compare the phenotype among different frameshift mutations. For obtaining *hoxa13a* ^+/-^,*hoxa13b* ^+/-^ double heterozygous mutant fish, each single heterozygous mutant line were crossed each other. Offspring were analyzed by T7E1 assay and sequenced after three months, and double heterozygous mutant fish were selected. For generating double homozygous *hoxa13* mutant embryos and adult fish (*hoxa13a*^-/-^, *hoxa13b*^-/-^), double heterozygous fish (*hoxa13a* ^+/-^, *hoxa13b* ^+/-^) were crossed each other. Ratio of each genotype from crossing heterozygous fish are summarized in Extended Data Table 4.

### Genotype of single (*hoxa13a* ^-/-^ or *hoxa13b* ^-/-^) or double (*hoxa13a* ^-/-^, *hoxa13b* ^-/-^) mutant by PCR

After establishing mutant lines, single (*hoxa13a* or *hoxa13b*) or double (*hoxa13a*, *hoxa13b*) mutant embryos and adult fish were genotyped by PCR for each analysis. Primer sequence for PCR are listed in Extended Data Table 2. To identify 8 bp deletion in exon1 of *hoxa13a*, PCR product was treated by Ava1 at 37 °C for two hours, because 8 bp deletion produces new Ava1 site in PCR product (“zebra *hoxa13a*_8 bp del” primers, wild-type; 231 bp, mutant; 111 bp + 119 bp). Final product size was confirmed by 3 % agarose gel electrophoresis. To identify 29 bp deletion in exon1 of *hoxa13a*, PCR product was confirmed by gel electrophoresis (“zebra *hoxa13a*_29 bp del” primers, wild-type; 110 bp, mutant; 81 bp). To identify 13 bp insertion in exon1 of *hoxa13b*, PCR product was treated by Bcc1 at 37 °C for two hours, because 13 bp insertion produces new Bcc1 site in PCR product (“zebra *hoxa13b*_13 bp ins” primers, wild-type; 98 bp, mutant; 53 bp + 57 bp). Final product size was confirmed by 3 % agarose gel electrophoresis. The detail of mutant sequence are summarized in Extended Data Table 3 a-c.

### Combination of stable and transient deletion of all *hox13* genes by CRISPR / Cas9

Two gRNAs targeting exon 1 of *hoxa13b* and two gRNAs targeting exon 1 of *hoxd13a* were injected with *Cas9* mRNA into zebrafish one cell eggs that were obtained from crossing *hoxa13a*^+/-^ and *hoxa13a*^+/-^, *hoxa13b*^+/-^, *hoxd13a*^+/-^ (gRNAs were same as that were used to establish single *hox13* knockout fishes and found in Extended Data Table 2). Injected eggs were raised to adult fish and genotyped by extracting DNA from tail fins. PCR products of each *hox13* gene were cloned into PCRIITOPO (Invitrogen) and deep sequencing was performed (Genomic Core, the University of Chicago). At four months old, skeletal staining and CT scanning were performed to analyze the effect of triple gene deletions. The knockout ratios of each *hox13* allele were calculated from the results of deep sequencing.

### Measurement of fin fold length

Embryos were obtained by crossing *hoxa13a* ^+/-^, *hoxa13b* ^+/-^ to each other and raised to 72 hpf or 96 hpf. After fixation by 4 % PFA for 15 hours, caudal halves were used for PCR genotype. Pectoral fins of wild-type and *hoxa13a* ^-/-^, *hoxa13b* ^-/-^ were detached from embryonic body and placed hotizontally on a glass slide. The fins were photographed with a Leica M205FA microscope, and the fin fold length along the proximodistal axis at the center of the fin was measured by ImageJ. The resulting data was subjected to a standard *t*-test.

### Counting cell number in endochondral disk

Embryos were obtained by crossing *hoxa13a* ^+/-^, *hoxa13b* ^+/-^ to each other and raised to 96 hpf. After fixation by 4 % PFA for 15 hours, caudal halves were used for PCR genotype. Wild-type and *hoxa13a* ^-/-^, *hoxa13b* ^-/-^ embryos were stained by DAPI (1/4000 in PBS-0.1 %Triton) for three hours and washed for three hours by PBS–0.1 % Triton. Pectoral fins were detached from embryonic body, placed on slide glasses and covered by a coverslip. DAPI signal was detected by Zeiss LSM 710 (Organismal Biology and Anatomy, the University of Chicago). Individual nuclei were manually marked using Adobe Illustrator and the number was counted. The data was subjected to a standard *t*-test.

### Adult fish skeletal staining

Skeletal staining was performed as previously described^33^. Briefly, fish were fixed by 10 % neutral-buffered formalin overnight. After washing by milli-Q water, solutions were substituted to 70 % EtOH in a stepwise fashion and then to 30 % acetic acid / 70 % EtOH. Cartilage were stained by 0.02 % alcian blue in 30 % acetic acid / 70 % EtOH overnight. Washed by milli-Q water, solution was changed to 30 % saturated sodium borate solution and incubated overnight. Then, specimens were immersed in 1 % trypsin / 30 % saturated sodium borate and incubated at room temperature overnight. Following milli-Q water wash, specimens were transferred into 1 % KOH solution including 0.005 % Alzarin Red S. Next day, specimens were washed by milli-Q water and had been subjected to glycerol substitution. Three replicates for each genotype were investigated.

### PMA staining and CT scanning

After skeletal staining, girdles and pectoral fins were manually separated from the body. Girdles and fins were stained by 0.5 % PMA (Phosphomolybdic Acid) in milli-Q water for 16 hours and followed by washes of milli-Q water. Specimens were placed into 1.5 ml eppendorf tubes with water and kept overnight to settle in the tubes. The next day, tubes containing specimens were set and scanned with the UChicago PaleoCT scanner (GE Phoenix v/tome/x 240kv/180kv scanner) (http://luo-lab.uchicago.edu/paleoCT.html), at 50 kVp, 160 μA, no filtration, 5x-averaging, exposure timing of 500 ms per image, and a resolution of 8.000 μm per slice (512 μm^3^ per voxel). Scanned images were analyzed and segmented using Amira 3D Software 6.0 (FEI). Three replicates for single and double homozygotes and five for mosaic triple knockout were investigated.

## Extended Data

**Extended Data Figure 1:**
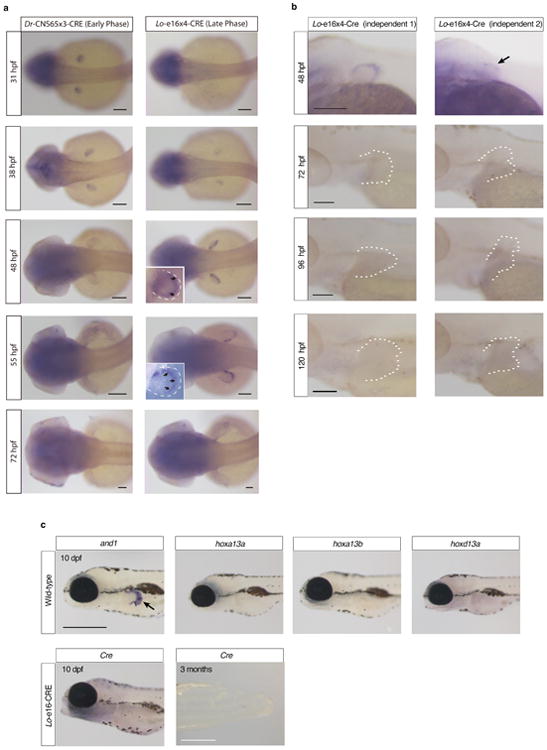
*Cre in situ* hybridization of lineage tracing fish **a.** *Cre* is expressed only from 31 hpf to 38 hpf in *Dr*-CNS65x3-Cre, whereas from 38 hpf to 55 hpf in *Lo*-e16x4-*Cre*. These temporal expression patterns of *Cre* indicate that our transgenic lineage tracing labeled the cells experienced only early or late phase *hox*. Scale bars are 100 μm. **b.** *Cre* expression pattern from 48–120 hpf in independent *Lo*-e16x4-Cre lines (different founders from **a**). The fin is outlined by a dashed white line. The expression patterns from different founders were investigated and all expression ceases before 72 hpf. Our *in situ* results, indicate that *Lo*-e16x4-Cre only marks the cells that experienced late phase *hox* expression from 38-55 hpf. *n* = 5 embryos for all stages. Scale bars are 100 μm. **c.** The expression pattern of *and1* and *hox13* genes in wild-type (10 dpf) and also *Cre* in *Lo*-e16x4-Cre line (10 dpf and 3 months, *n* = 10). Whereas *and1* expression can be observed in fin fold (positive control, black arrow), *hox13* genes are not expressed at 10 dpf in wild-type. *Cre* is not expressed at 10 dpf and 3 months fin, indicating that *Lo*-e16x4-Cre activity is limited only early embryonic development (38-55 hpf). 3 month fins were dissected from the body of *Lo*-e16x4-Cre lines and subjected to *in situ* hybridization (*n* = 3). Scale bars are 500 μm at 10 dpf and 3 months.

**Extended Data Figure 2:**
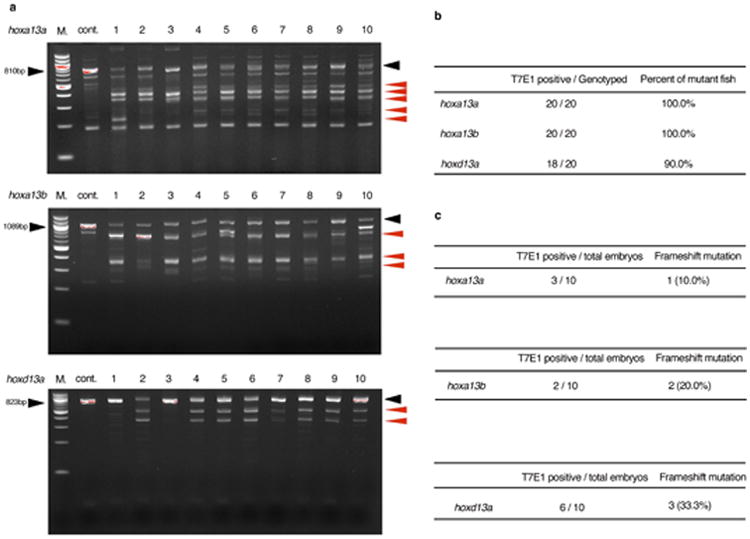
T7E1 assay of F0 CRISPR/Cas9 adult fish PCR products of each *hoxa13a*, *hoxa13b* or *hoxd13a* were subjected to T7E1 assay (Methods) and confirmed by gel electrophoresis. **a**. the result of *hoxa13a, hoxa13b or hoxd13a* T7E1 assay for ten adult fish. “M.” is a 100 bp DNA ladder marker (NEB). In the *hoxa13a* gel picture, 810 bp (black arrowhead) is wild-type band as observed in cont. lane (wild-type without gRNAs injection). All ten fish showed the smaller and bottom shift products (red arrowheads) compared with negative control fish, indicating that all fish have mutations in the target region of *hoxa13a*. In *hoxa13b* gel picture, 1089 bp is wild-type band. All ten fish into which *hoxa13b* gRNAs were injected showed the smaller and bottom shift products compared with negative control fish, indicating that all fish have mutations in the target region of *hoxa13b*. In *hoxd13a* gel picture, 823 bp is wild-type band. Eight of ten fish showed the smaller and bottom shift products, indicating that 80 % fish have mutations in the target region of *hoxd13a*. **b**, The efficiency of CRISPR/Cas9 deletion for *hox13* in zebrafish. Almost all adult fish into which gRNAs and *Cas9* mRNA were injected have mutations at the target positions. **c**, The efficiency of germline transmission of CRISPR/Cas9 mutant fish. Identified mutant fish were outcrossed to wild-type to obtain embryos and confirmed germline transmission. Obtained embryos were lysed individually at 48 hpf, genotyped by T7E1 assay and sequenced. Because of CRISPR/Cas9 mosaicism, some different mutation patterns, that result in a non-frameshift or frameshift mutation, were observed.

**Extended Data Figure 3:**
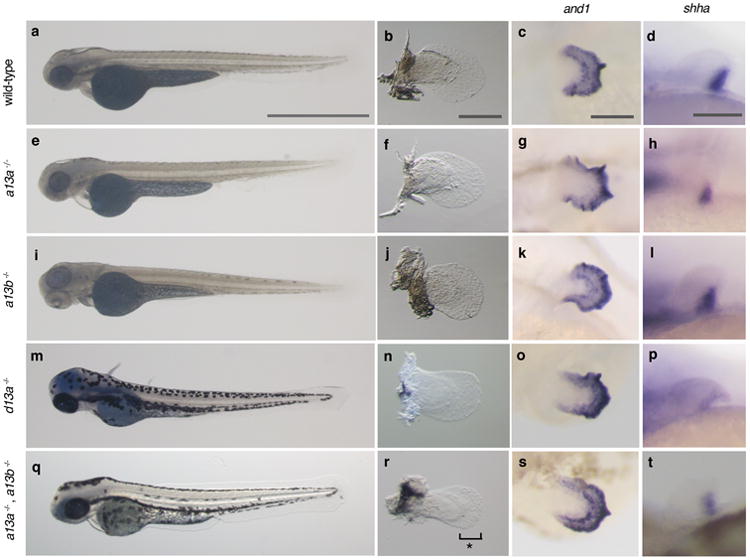
Embryonic phenotypes of *hox13* deletion mutants **a**, **e**, **i**, **m**, **q**, Whole body pictures at 72 hpf; *hoxa13a*^-/-^ (4 bp del. / 4 bp del.), *hoxa13b*^-/-^ (4 bp del. / 13 bp ins.), *hoxd13a*^-/-^ (5 bp ins. / 17 bp del.), and *hoxa13a*^-/-^, *hoxa13b*^-/-^ double homozygous embryo (8 bp del. / 29 bp del., 13 bp ins. / 13 bp ins.). The detail of mutant sequence is summarized in Extended Data Table 3. Wild-type and single homozygous fish of *hoxa13a* or *hoxa13b* were treated by PTU to inhibit pigmentation. The body size and length of mutant embryos are relatively normal at 72 hpf. *n* = 5 embryos for all genotypes. **b**, **f**, **j**, **n**, **r**, Bright field images of pectoral fins. Pectoral fins were detached from the body and photographed (Methods). *Hoxa13a* ^-/-^, *a13b*^-/-^ double homozygous embryo shows 30 % shorter pectoral fin fold compared with wild-type (**r**, see also Extended Data Fig. 4). *n* = 5 embryos for all genotypes. **c**, **g**, **k**, **o**, **s**, *and1 in situ* hybridization at 72 hpf. *Hox13* mutants show normal expression patterns, which indicates that fin fold development is similar to wild-type in these mutants. *n* = 3 embryos for all genotypes. **d**, **h**, **l**, **p**, **t**, *shha in situ* hybridization at 48 hpf. *Hox13* mutants show a normal expression pattern that is related to relatively normal anteroposterior asymmetry of adult fin (Figure 3, Extended Data Fig. 5, Supplementary Information). *n* = 3 embryos for all genotypes. Scale bars are 1 mm (**a**), 200 μm (**b**,**c**) and 100 μm (**d**).

**Extended Data Figure 4:**
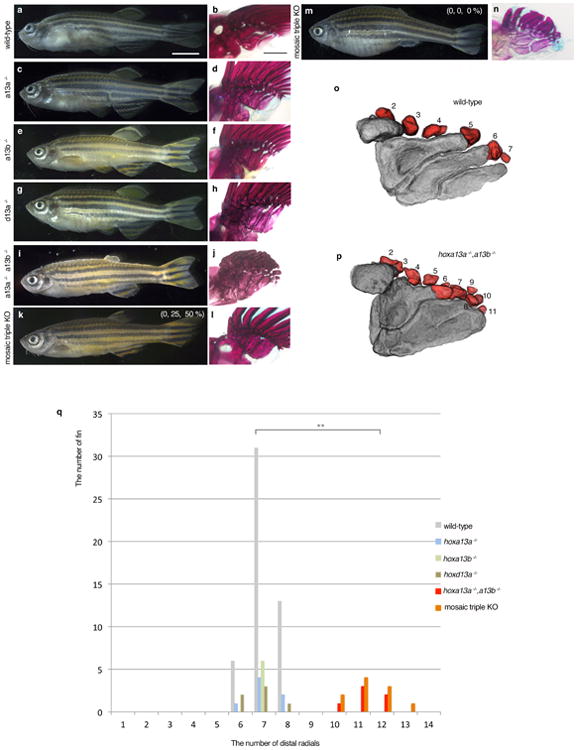
Analysis of embryonic fin fold and endochondral disk in *hoxa13a*^-/-^, *hoxa13b*^-/-^ embryo **a**. A bright field image of wild-type and *hoxa13a*^-/-^, *hoxa13b*^-/-^ pectoral fins at 72 hpf. Pectoral fins were detached from the body and photographed (Methods). Scale bar is 150 μm. **b**, The difference of the fin fold length in wild-type and *hoxa13a*^-/-^, *hoxa13b*^-/-^ embryos. The length of the fin fold were measured in wild-type (*n*=8) and *hoxa13a*^-/-^, *hoxa13b*^-/-^ double homozygous (*n*=5) embryos at 72 hpf and 96 hpf (Methods). The length of the fin folds were decreased to about 70% of wild-type in double homozygous embryos (72 hpf; *p*=0.006, 96 hpf; *p*=0.004 by compare means t-test, one-tailed distribution, See Source Data). The error bars indicate Standard Error. **c**,**d**, Images of DAPI staining of wild-type (**c**) and *hoxa13a*^-/-^, *hoxa13b*^-/-^ mutant (**d**) pectoral fins captured by the confocal microscope. White circles indicate nuclei in the endochondral disks. Scale bar is 200 μm. **e**, the average number of cells in the endochondral disk of wild-type and *hoxa13a*^-/-^, *hoxa13b*^-/-^ mutant fins (See Methods and Source data also). The difference is statistically significant (*p* = 0.041 by compare means t-test, one-tailed distribution). The error bars indicate Standard Error.

**Extended Data Figure 5:**
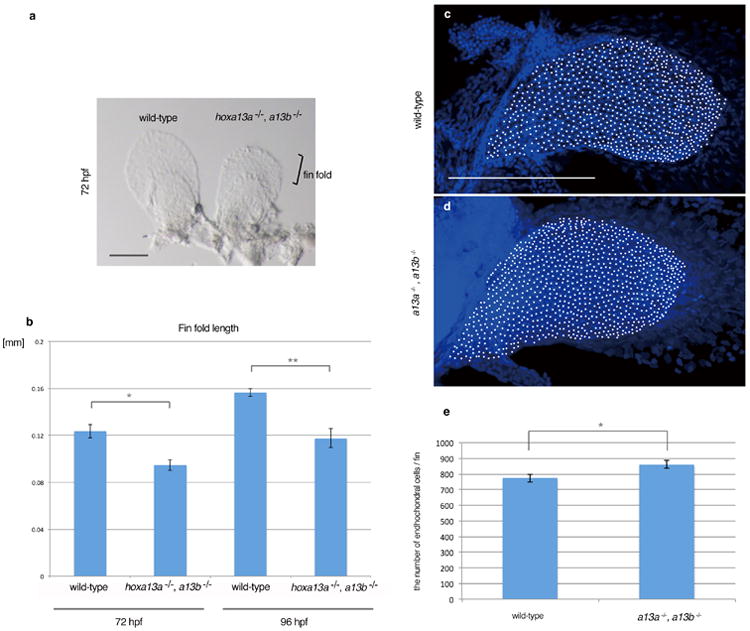
Phenotype of adult *hox13* mutant fish **a**, **c**, **e**, **g**, **i**, **k**, **m**. Whole body morphology of *hox13* deletion mutants were photographed at four months old; *hoxa13a*^-/-^ (8 bp del. / 29 bp del.), *hoxa13b*^-/-^ (4bp del. / 13 bp ins.), *hoxd13a*^-/-^ (5 bp ins. / 10 bp ins.), *hoxa13a*^-/-^, *hoxa13b*^-/-^ double homozygous fish (8 bp del. / 29 bp del., 13bp ins. / 13 bp ins.) and triple knockout (**k**, **m**. mosaic for *hoxa13b* and *hoxd13a*) fish (Methods). *n* = 3 fish for wild-type, single and double mutants and *n* = 5 fish for triple mosaic mutants (same specimens were used as Figure 3). The detail of mutant sequence is summarized in Extended Data Table 3. Each single homozygous mutant fish shows normal morphology at four months old except for slightly short pectoral fin rays of *hoxa13a*^-/-^ or *a13b*^-/-^ single mutant. *Hoxa13a*^-/-^, *hoxa13b*^-/-^ double homozygous fish shows a severe reduction of fin rays in pectoral, pelvic, dorsal and anal fins compared with wild-type. The triple knockout (mosaic for *hoxa13b* and *hoxd13a*) fish also showed the reduction of fin rays. Scale bar is 5 mm. Due to the size of the adult fish, three different pictures for anterior, center and posterior of the body were merged to make whole body pictures. **b**, **d**, **f**, **h**, **j**, **l**, **n**, Bone staining pictures of mutant fish. The endochondral bones of pectoral fins are shown. Whereas single homozygous fish show relatively normal proximal radials (**b**, **d**, **f**, **h**, Figure 3), double homozygous mutants show fused third and fourth proximal radials (**j**). One of triple knockout (mosaic for *hoxa13b* and *hoxd13a*, 0, 25, 50 %) fish show fused third and fourth proximal radials (**i**), but another triple knockout (0, 0, 0 %) show more broken down proximal radials (**n**). *n* = 3 fish for wild-type, single and double mutants and *n* = 5 fish for triple mosaic mutants (same specimens were used as Figure 3). The scale bar is 500 μm. **o**, **p**, Examples of counting distal radials in wild-type and *hoxa13a*^-/-^, *hoxa13b*^-/-^ double homozygous fish. First distal radials are not shown in CT segmentation because of a fusion with first fin ray. **q**, The number variation of distal radial in mutant fish. Multiple fins were investigated in wild-type (25 fish / 50 fins), *hoxa13a*^-/-^ (4 bp del. / 4 bp del., three fish / 6 fins), *hoxa13b*^-/-^ (4 bp del. / 13 bp ins., three fish / 6 fins), *hoxd13a*^-/-^ (5 bp ins. / 17 bp del., three fish / 6 fins), *hoxa13a*^-/-^, *hoxa13b*^-/-^ double homozygous (8 bp del. / 29 bp del., 13 bp ins. / 13 bp ins., three fish / 6 fins) and triple knockout (mosaic for *hoxa13b* and *hoxd13a*) fish (five fish / 10 fins). The number of distal radials increased to 10 to 13 in double and triple mutants. The difference of distal radial number between wild-type and double homozygous or wild-type and triple knockout (mosaic for *hoxa13b* and *hoxd13a*) is statistically significant (*p*= 0.0014 or *p* = 0.00001 by compare means t-test, two-tailed distribution).

**Extended Data Table 1:** Primers and oligos sequence for lineage tracing PCR primers and oligos for construction of lineage tracing vectors are listed (See Methods also). Restriction enzyme sites that were used for ligating oligos are highlighted by italic and bold in oligo sequence.

Lineage tracing oligos
CRE_PCR_F_NcoI
5′- CGCCCTTCCATGGATGGCCAATTTACTGACCGTAC -3′
CRE_PCR_R_BglII
5′- GTTCTTCTGAAGATCTCTCTGGGGTTCGGGGCTGCAGG -3′
CRE_Genotype_F
5′- CGTACTGACGGTGGGAGAAT -3′
CRE_Genotype_R
5′- ACCAGGCCAGGTATCTCTGA -3′
CRE_Probe_F
5′- ATGGCCAATTTACTGACCGTAC -3′
CRE_Probe_R
5′- CTAATCGCCATCTTCCAGCAGGCG -3′
Random_Oligo_Smal
5′- CTGCTCTGGTCAGCCTCTAATGGCTCGTTAGATAGTCTAGCCGCTGGTAATCACTCGATGACCTCGGCTCCCCATTGGTGCTACGGCGATTCTTGGAGAGCCAGCTGCGATCGCTAATGTGAGGACAGTGTAATATTAG CAAGCGATAAGTCCCCAACTGGTTGTGGCCTTTTGAAAAGTGAACTTCATAACATATGCTGTCTCACGCACATGGATGGTTTGGACAAATTTGATTCAAGTCTGATCAACCTTCACTGCTCTAGAATCAAAAGCAGTGATCTC CCGGGTGCGAAATAAA -3′ Smal site italicized in bold
Lo-e16_Oligo_1_BamHI_Smal
5′- CCCCCAAAAAATGACAAAACTCTTGGAATTTATTACGGCTTTGGCAATAGAGACCGCTTTTTGGGTGGCTCAGTAAAAGGTTTGATGTTCACGTATCGCCTTTTAAATGCATTCATTCCTCTTTCATATGTGTGCAACTGTT TAGATACATCATAAAAATGTCACCATTGAGGTTCCCCATTAGGCATCTACCCGTTCTCCTCCAGGCCATGGAGATAAATTTGGACCAGGTGATCCCCTCCTAGAAGAGCCCTTGATGTCTTCTGGTAATGAGTTGAAAGCGGA AGCTGTCAGCCTTCAGCAGGCATGAAGATGCAATTAGAGCTGCGTTCAAAGTGCCCAGGCAGTCTCATAAGGAGCACTAGCCTTGGTGTAAGCTGCTTATTCACAGATCAGTTATGTAAGGGTACAGCAAAAAGGCAAGAC ACTCGATTTTTGAATGACACAGCAAAGTCGGTGCGGATCCCGAGTTTGCCCGGGTAGCCC -3′ BamHI and Smal sites italicized in bold
Lo-e16_Oligo_2_BamHI_SalI_Smal
5′- CCCCCAAAAAATGACAAAAGGATCCGAATTTATTACGGCTTTGGCAATAGAGACCGCTTTTTGGGTGGCTCAGTAAAAGGTTTGATGTTCACGTATCGCCTTTTAAATGCATTCATTCCTCTTTCATATGTGTGCAACTGTTT AGATACATCATAAAAATGTCACCATTGAGGTTCCCCATTAGGCATCTACCCGTTCTCCTCCAGGCCATGGAGATAAATTTGGACCAGGTGATCCCCTCCTAGAAGAGCCCTTGATGTCTTCTGGTAATGAGTTGAAAGCGGAAG CTGTCAGCCTTCAGCAGGCATGAAGATGCAATTAGAGCTGCGTTCAAAGTGCCCAGGCAGTCTCATAAGGAGCACTAGCCTTGGTGTAAGCTGCTTATTCACAGATCAGTTATGTAAGGGTACAGCAAAAAGGCAAGACACT CGATTTTTGAATGACACAGCAAAGTCGTCGACTTCTCCGAGCCCGGGAAACTAGCCC -3′ BamHI, SalI, and Smal sites italicized in bold
Lo-e16_Oligo_3_SalI_BglII_Smal
5′- CCCCCAAAAAATGACGTCGACCTTGGAATTTATTACGGCTTTGGCAATAGAGACCGCTTTTTGGGTGGCTCAGTAAAAGGTTTGATGTTCACGTATCGCCTTTTAAATGCATTCATTCCTCTTTCATATGTGTGCAACTGTT TAGATACATCATAAAAATGTCACCATTGAGGTTCCCCATTAGGCATCTACCCGTTCTCCTCCAGGCCATGGAGATAAATTTGGACCAGGTGATCCCCTCCTAGAAGAGCCCTTGATGTCTTCTGGTAATGAGTTGAAAGCGGAA GCTGTCAGCCTTCAGCAGGCATGAAGATGCAATTAGAGCTGCGTTCAAAGTGCCCAGGCAGTCTCATAAGGAGCACTAGCCTTGGTGTAAGCTGCTTATTCACAGATCAGTTATGTAAGGGTACAGCAAAAAGGCAAGACAC TCGATTTTTGAATGACACAGCAAAGTCGAGATCTTCTCCGAGTCCCGGGAACTAGCCC -3′ SalI, BglII, and Smal sites italicized in bold
Lo-e16_Oligo_4_BglII_Smal
5′- CCCCCAAAAAATGAGATCTCTCTTGGAATTTATTACGGCTTTGGCAATAGAGACCGCTTTTTGGGTGGCTCAGTAAAAGGTTTGATGTTCACGTATCGCCTTTTAAATGCATTCATTCCTCTTTCATATGTGTGCAACTGTTT AGATACATCATAAAAATGTCACCATTGAGGTTCCCCATTAGGCATCTACCCGTTCTCCTCCAGGCCATGGAGATAAATTTGGACCAGGTGATCCCCTCCTAGAAGAGCCCTTGATGTCTTCTGGTAATGAGTTGAAAGCGGAAG CTGTCAGCCTTCAGCAGGCATGAAGATGCAATTAGAGCTGCGTTCAAAGTGCCCAGGCAGTCTCATAAGGAGCACTAGCCTTGGTGTAAGCTGCTTATTCACAGATCAGTTATGTAAGGGTACAGCAAAAAGGCAAGACACT CGATTTTTGAATGACACAGCAAAGTCCCCGGGTTCTCCGAGAAACTAGCCC -3′ BglII, and Smal sites italicized in bold
Primers for final PCR to clone into destination vector:
e16x4_F_Xho1:
5′- CAGGCTCCCTCGAGCCCCCAAAAAATGACAAA -3′
e16x4_R_Smal:
5′- CGAATTCGGTCCCGGGACTTTGCTG -3′
Dr-CNS65_Oligo_1_BamHI_Smal
5′- GAGGTTCACCTTTAACCACAACACGTAACAAATCAGATCTCAGAAGACAAGCCGCTTCAGAAGTCGTGCTCAGTGTTGCATTCAAGCGTGTGTGATTTTCCAGACTGTCTGTGTGTGTGTGTGTGTGTGTGTGTGTGTGT GTGTGTGTGTGTGTGCTCTCAGAGATCTTTCATTGGGGAATCTTTCCTGTGTGAGAGCTGCGGTCTCAGCGGCTGATTTATGGCGCTCCGCAGCTATGCTCATGCTACGCTAACAATGCTCATTAAAAAGAGGATGTCATCAC TCCGCGACACCGCAGGACTCGTATGTGTCACATGCATCCTCAATACAGCGAACCGCTGACCAATACCGTCCACAACATCCTGTAAATCTGTCATCGCCAGCATGGCCGCGGAAACACACACACACACACACACCATTAGAGTG CAGTAATAGAGGATCAGAGGTTAATGTGGAGCTGTTTGCTGGTGTTTAGTTTTGTATTAGAGGATTTCACGTGCTTACAGCTATGTGTGTGTGTTTGAACAGTAAAGAAAGTATAAAAAGTAAAATATTATAATCTTAAGCCACTCG TAATCTTCAAAAAACACTAAAATGCAAGAATAACGGATCCCTTTCACACTAGAGCCCGGGAAAGTGAGCGTT -3′ BamHI and Smal sites italicized in bold
Dr-CNS65_Oligo_2_BamHI_SalI_Smal
5′- GAGGTTCACCTTTAGGATCCACACGTAACAAATCAGATCTCAGAAGACAAGCCGCTTCAGAAGTCGTGCTCAGTGTTGCATTCAAGCGTGTGTGATTTTCCAGACTGTCTGTGTGTGTGTGTGTGTGTGTGTGTGTGTGT GTGTGTGTGTGTGTGCTCTCAGAGATCTTTCATTGGGGAATCTTTCCTGTGTGAGAGCTGCGGTCTCAGCGGCTGATTTATGGCGCTCCGCAGCTATGCTCATGCTACGCTAACAATGCTCATTAAAAAGAGGATGTCATCAC TCCTGATTTATGGCGCTCCGCAGCTATGCTCATGCTACGCTAACAATGCTCATTAAAAAGAGGATGTCATCACTCCGCGACACCGCAGGACTCGTATGTGTCACATGCATCCTCAATACAGCGAACCGCTGACCAATACCGTCC ACAACATCCTGTAAATCTGTCATCGCCAGCATGGCCGCGGAAACACACACACACACACACACCATTAGAGTGCAGTAATAGAGGATCAGAGGTTAATGTGGAGCTGTTTGCTGGTGTTTAGTTTTGTATTAGAGGATTTCACGT GCTTACAGCTATGTGTGTGTGTTTGAACAGTAAAGAAAGTATAAAAAGTAAAATATTATAATCTTAAGCCACTCGTAATCTTCAAAAAACACTAAAATGCAAGAATAAGTCGACCCTTTCACACTAGGCCCGGGAAAGTGAGCGT -3′ BamHI, SalI, Smal sites italicized in bold
Dr-CNS65_Oligo_3_SalI_Smal
5′- GAGGTTCACCTTTAGTCGACACACGTAACAAATCAGATCTCAGAAGACAAGCCGCTTCAGAAGTCGTGCTCAGTGTTGCATTCAAGCGTGTGTGATTTTCCAGACTGTCTGTGTGTGTGTGTGTGTGTGTGTGTGTGTGT GTGTGTGTGTGTGTGCTCTCAGAGATCTTTCATTGGGGAATCTTTCCTGTGTGAGAGCTGCGGTCTCAGCGGCTGATTTATGGCGCTCCGCAGCTATGCTCATGCTACGCTAACAATGCTCATTAAAAAGAGGATGTCATCAC TCCGCGACACCGCAGGACTCGTATGTGTCACATGCATCCTCAATACAGCGAACCGCTGACCAATACCGTCCACAACATCCTGTAAATCTGTCATCGCCAGCATGGCCGCGGAAACACACACACACACACACACCATTAGAGTG CAGTAATAGAGGATCAGAGGTTAATGTGGAGCTGTTTGCTGGTGTTTAGTTTTGTATTAGAGGATTTCACGTGCTTACAGCTATGTGTGTGTGTTTGAACAGTAAAGAAAGTATAAAAAGTAAAATATTATAATCTTAAGCCACTC GTAATCTTCAAAAAACACTAAAATGCAAGAATAACCCTTTCACACTAGAGCCCGGGAAAGTGAGCGTT -3′ SalI and Smal sites italicized in bold
Primers for final PCR to clone into destination vector:
CNS65x3_F_XhoI:
5′- GCAGGCTCCTCGAGGAGGTTCACCTTTAACCA -3′
CNS54x3_R_Smal:
5′- AACGCTCACTTTCCCGGGTCTAGTGT -3′

**Extended Data Table 2:** PCR primers for CRISPR/Cas9 deletion, T7E1 assay, genotypes and gene cloning For synthesis of gRNAs, each forward primer and common reverse primer (“zebra gRNA_R”) were hybridized and used as templates. For genotype of single and double mutants, PCR products were treated by the enzymes indicated.

CRISPR gRNA oligos	
zebra hoxa13a_gRNA1_F	
5′- AATTAATACGACTCACTATAGGGCAATCACAACCAGTGGAGTTTTAGAGCTAGAAATAGC -3′	
zebra hoxa13a_gRNA2_F	
5′- AATTAATACGACTCACTATAGGCAGTAAAGACTCATGTCGGTTTTAGAGCTAGAAATAGC -3′	
zebra hoxa13b_gRNA1_F	
5′- AATTAATACGACTCACTATAGGATGATATGAGCAAAAACAGTTTTAGAGCTAGAAATAGC -3′	
zebra hoxa13b_gRNA2_F	
5′- AATTAATACGACTCACTATAGGACACTTCTGTTTCTGGAGGTTTTAGAGCTAGAAATAGC -3′	
zebra hoxd13a_gRNA1_F	
5′- AATTAATACGACTCACTATAGGCTCTGGCTCCTTCACGTTGTTTTAGAGCTAGAAATAGC -3′	
zebra hoxd13a_gRNA2_F	
5′- AATTAATACGACTCACTATAGGCGAACTCTTTAAGCCAGCGTTTTAGAGCTAGAAATAGC -3′	
zebra gRNA_R	
5′- AAAAGCACCGACTCGGTGCCACTTTTTCAAGTTGATAACGGACTAGCCTTATTTTAACTTGCTATTTCTAGCTCTAAAAC -3′	
T7 assay primers	Genotype primers for single (hoxa13a or a13b) and double (hoxa13a, a13b) mutants
zebra hoxa13a_Cont_F	zebra hoxa13a_8 bp del_F
5′- CTGCAGCGGGTGATTCTG -3′	5′- GCCAAGGAGTTTGCCTTGTA -3′
zebra hoxa13a_Cont_R	zebra hoxa13a_8 bp del_R
5′- CTCCTTTACCCGTCGGTTTT -3′	5′- TGACGACTTCCACACGTTTC -3′
PCR product: 810 bp	PCR product: wild-type 231 bp, mutant (cut by Ava1) 111 +119 bp
zebra hoxa13b_Cont_F	zebra hoxa13a_29 bp del_F
5′- GAAGCTTATCACTAGAATCTTTACAGC -3′	5′- CAGGCAATAAGCGGGCCTT -3′
zebra hoxa13b_Cont_R	zebra hoxa13a_29 bp del_R
5′- TTTTTCTCAGGGCCTAAAGGT -3′	5′- GTGCAGTAGACCTGTCCGTT -3′
PCR product:1089 bp	PCR product: wild-type 110 bp, mutant 81 bp
zebra hoxd13a_Cont_F	zebra hoxa13b_13 bp ins_F
5′- AGCTGCCCAATCACATGC -3′	5′- TACACTGGTTCGCAGCAAAA -3′
zebra hoxd13a_Cont_R	zebra hoxa13b_13 bp ins_R
5′- CGATTATAAATTCAGTTGCTCTTTAG -3′	5′- GATTGACCCGGTGATGTTTC -3′
PCR product: 823 bp	PCR product: wild-type 98 bp, mutant (cut by Bcc1) 53 + 57 bp
Cloning primers	
Danio_and1_F	
5′-ACCTGCTCCTGCTCCAGTTA -3′	
Danio_and1_R	
5′- CACATCCTCTTGAGGGGAAA -3′	

**Figure 3:**
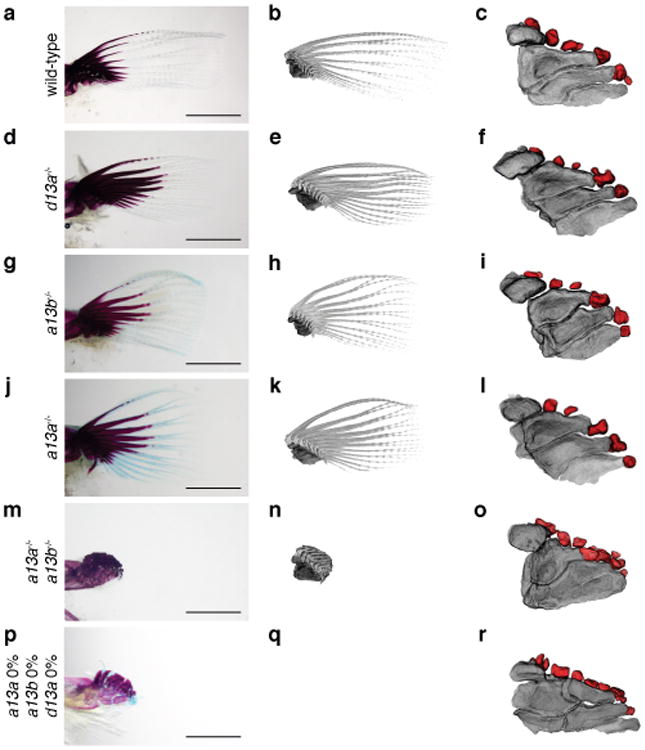
Adult fin phenotypes of *hox13* deletion series **a-c**, wild-type. **d-f**, *hoxd13a*^-/-^. **g-i**, *hoxa13b* ^-/-^. **j-l**, *hoxa13a*^-/-^. **m-o**, *hoxa13a*^-/-^, *a13b*^-/-^. **p-r** *hoxa13a*^0%^ *a13b*^0%^ and *d13a ^0%^* (mosaic triple knockout; Methods and Extended Data Table 3,4). Each mutant *hox* sequence is found in Extended Data Table 3,4. **a**, **d**, **g**, **j**, **m**, **p**, Alzarin Red and Alcian Blue staining of pectoral fin. **b**, **e**, **h**, **k**, **n**, **q**, CT scanning of pectoral fins. Black: radials (endochondral bones); gray: fin rays (dermal bones). Note that *hoxa13* single (**g**, **h**, **j**, **k**), double (**m**, **n**), and mosaic triple (**p**, **q**) mutant fins show shorter fin rays than wild-type (**a**, **b**). Fins were scaled according to the bone staining pictures. **c**, **f**, **i**, **l**, **o**, **r**, Enlarged images of CT scanning without fin rays to reveal endochondral patterns. Dark gray; proximal radials, red; distal radials. Upper left side is the anterior and bottom right is the posterior side in each pictures. Double and triple knockout mutants have 10-13 distal radials (**o** and, **r**, Extended Data Fig. 5, Supplementary Information). Third and fourth proximal radials started to fuse into one bone in *hoxa13a*^-/-^, *a13b*^-/-^ (**o**). Note that posterior distal radials are stacked along proximodistal axis (**o**). Posterior proximal radials are broken down into small parts in mosaic triple knockout (**r**). Scale bars are 2 mm. The size of specimens are not scaled in **c**, **f**, **i**, **l**, **o** and **r** to display the detail of distal radials. *n* = 3 fish for single and double mutants and *n* = 5 fish for mosaic triple mutant.

**Extended Data Table 3:** List of *hox13* mutant sequences Frame shift mutation alleles that were used for each experiment are listed. The top sequence in each column show wild-type with gRNA sequence in red. Green is insertional, blue is substitutional mutations. **a**. *hoxa13b* mutation patterns. Sequence flanked by two gRNAs are abbreviated by black horizontal bars. **c**. *hoxd13a* mutation patterns. Sequence flanked by two gRNAs are abbreviated by black horizontal bars. **d-f**, Mutational patterns in triple knockout (mosaic for *hoxa13b* and *hoxd13a*) fish that is shown in Figure 3p-r are listed. Sequence flanked by two gRNAs are abbreviated by horizontal bars in **e** and **f**. Each *hox13* gene shows some different mutations indicating that this fish is highly mosaic. The percentage of mutant alleles were calculated from the result of deep sequencing (Figure 3, Extended Data Table 4). **g.** Summary of genotype in all experiments. del. (deletion) and ins. (insertion)

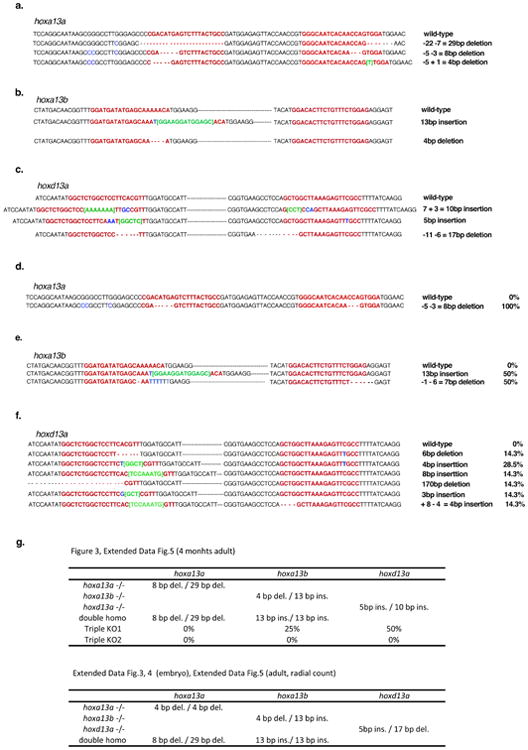

**Extended Data Table 4:** **a**, Breeding data in *hox13* single mutants. Single heterozygous fish were crossed each other to obtain embryos and next generations. Embryos (72 hpf) or adult fish (three months) were genotyped by T7E1 assay and sequenced. The number of each genotype and percentages are shown. The ratio of each genotype approximately follows Mendelian ratio. **b**, Breeding data of double *hoxa13* mutants. Double heterozygous fish (*hoxa13a*^+/-^, *hoxa13b* ^+/-^) were crossed to obtain embryos and next generations. Embryos (72 hpf) or adult fish (three months) were genotyped by PCR followed by enzyme digestions (Methods) or sequencing. The number of each genotype and percentage are shown. The ratio of each genotype approximately follows Mendelian ratio. **c**, The efficiency of triple knockout (mosaic for *hoxa13b* and *hoxd13a*) in zebrafish (See Methods also). The number of normal adult fish and short finned fish from negative control injection (*Cas9* mRNA without gRNAs) or triple knockout injection (*Cas9* mRNA with gRNAs) are shown. Genotypes for short finned fish were calculated from deep sequencing of each allele and shown as a percentage of normal alleles in **d**.

a				
*hoxa13a*^+/-^ × *hoxa13a*^+/-^				

	+/+	+/-	-/-	Total
Embryos (72 hpf)	9 (25.0%)	17 (47.2%)	10 (27.8%)	36
Adult	9 (21.4%)	20 (47.6%)	13 (31%)	42

*hoxa13b*^+/-^ *× hoxa13b*^+/-^				

	+/+	+/-	-/-	Total

Embryos (72 hpf)	8 (25.0%)	20 (62.5%)	12 (37.5%)	32
Adult	20 (32.3%)	32 (51.6%)	10 (16.1%)	62

*hoxd13a*^+/-^ *× hoxd13a*^+/-^				

	+/+	+/-	-/-	Total

Embryos (72 hpf)	8 (22.9%)	18 (51.4%)	9 (25.7%)	35
Adult	5 (26.3%)	11 (57.9%)	3 (15.8%)	19

b				
*hoxa13a*^+/-^*, a13b*^+/-^ × *hoxa13a*^+/-^*, a13b*^+/-^ (72hpf)				

*a13b*	+/+	+/-	-/-	Total
*a13a*				
+/+	20 (11.0%)	25 (13.7%)	10 (5.5%)	55 (30.2%)
+/-	23 (12.6%)	50 (27.5%)	10 (5.5%)	83 (45.6%)
-/-	6 (3.3%)	28 (15.4%)	10 (5.5%)	44 (24.2%)

Total	49 (26.9%)	103 (56.6%)	30 (16.5%)	182 (100.0%)

*hoxa13a*^+/-^*, a13b*^+/-^ *× hoxa13a*^+/-^*, a13b*^+/-^ (Adult)				

a13b	+/+	+/-	-/-	Total
a13a				

+/+	4	8	0	12 (22.2%)
+/-	4	18	5	27 (50.0%)
-/-	3	5	7	15 (27.8%)

Total	11 (20.4%)	31 (57.4%)	12 (22.2%)	54 (100.0%)

c			

	total adulf fish	short finned fish	%
Negative control: *Cas9* only	96	0	0.00
*Cas9,hoxa 13b* and *d13a* gRNAs	161	7	4.35

d							
Genotype of short fin fish (The percent of normal alleles are shown)							

	#1	#2	#3	#4	#5	#6	#7
*hoxa13a*	20%	50%	0%	0%	25%	25%	0%
*hoxa13b*	20%	0%	25%	0%	0%	0%	0%
*hoxd13a*	100%	67%	50%	25%	30%	100%	0%

## Supplementary Material

supp_info

## Figures and Tables

**Figure 1:**
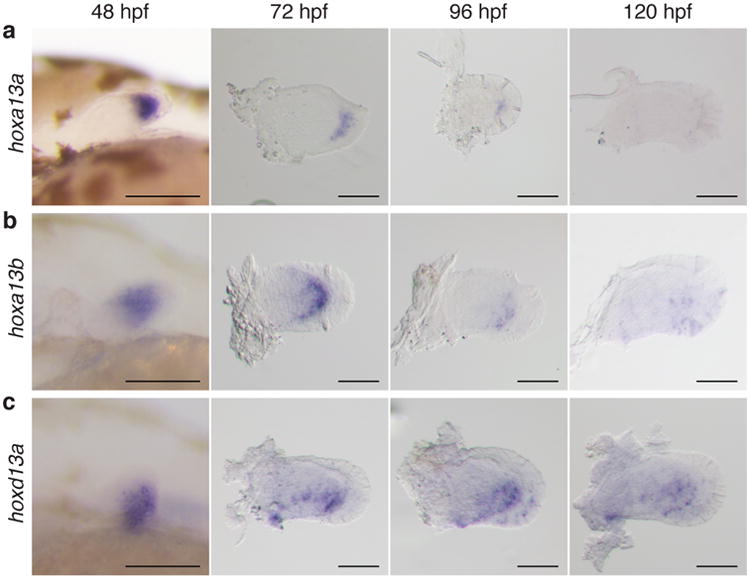
Expression patterns of *hox13* genes from 48-120 hpf. **a**, *hoxa13a.* **b**, *hoxa13b.* **c**, *hoxd13a.Hoxa13a* is expressed in distal mesenchyme at 48 hpf, but expression continues in the proximal fin fold from 72 to 96 hpf (**a**). *Hoxa13b* is expressed in distal mesenchyme and expression can be observed at the distal part of the endochondral disk until 96 hpf (**b**). *Hoxd13a* is expressed in the posterior half of the mesenchyme at 48 hpf and expression continues in the posterior endochondral disk through 96 hpf. After 96 hpf, expression becomes weak (**c**). Scale bars are 100 μm. *n* = 20 embryos for each *in situ* hybridization at 48 hpf. *n* = 10 embryos after 72 hpf.

**Figure 2:**
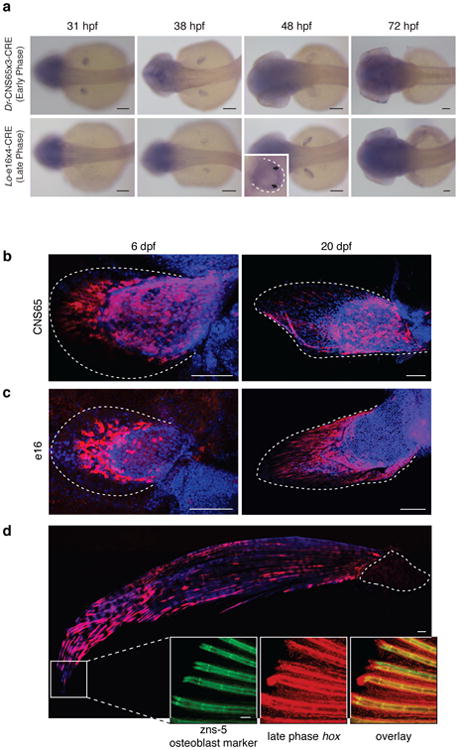
Fate mapping of cells marked by the activity of *hox* enhancers. **a**, *In situ* hybridization of *Cre* in *Dr*-CNS65x3-Cre and *Lo*-e16x4-Cre exhibits expression dynamics of early and late phase enhancers used for fate mapping. *Cre* regulated by early phase *hox* enhancer CNS65 is expressed throughout the fin from 31-38 hpf, whereas late phase expression (driven by e16) begins weakly in the distal fin at 38 hpf and ceases at ∼55 hpf. Black arrows point to the distal border of the endochondral disk. **b**, Lineage tracing of *Dr*-CNS65x3-Creat 6 dpf and 20 dpf. Red: mCherry IF; blue: DAPI. Cells that experienced early phase expression (red) contribute to fin fold and endochondral disk. **c**, Lineage tracing of *Lo*-e16x4-Cre at 6 dpf and 20 dpf. Cells that received late phase expression are present mostly in the fin fold, though some cells are at the distal edge of the disk. Red cells at 6 dpf protrude filopodia in the distal direction, indicating that these cells are actively moving out into the fin fold. **d**, Lineage tracing of late phase *hox* cells in adult zebrafish fin (∼120 dpf). mCherry cells are present only in the derivatives of the fin fold, and not in the endochondral disk. Inset: magnification of distal edge of fin rays. Green: Zns-5 osteoblast marker; red: Hox-positive; yellow: overlap of zns5 and Cre. White dotted lines outline the fin (**c**, **d**) or endochondral bones (**d**). *n* = 5 for stable lines. All scale bars are 100 um except for d, which is 500 um

**Figure 4:**
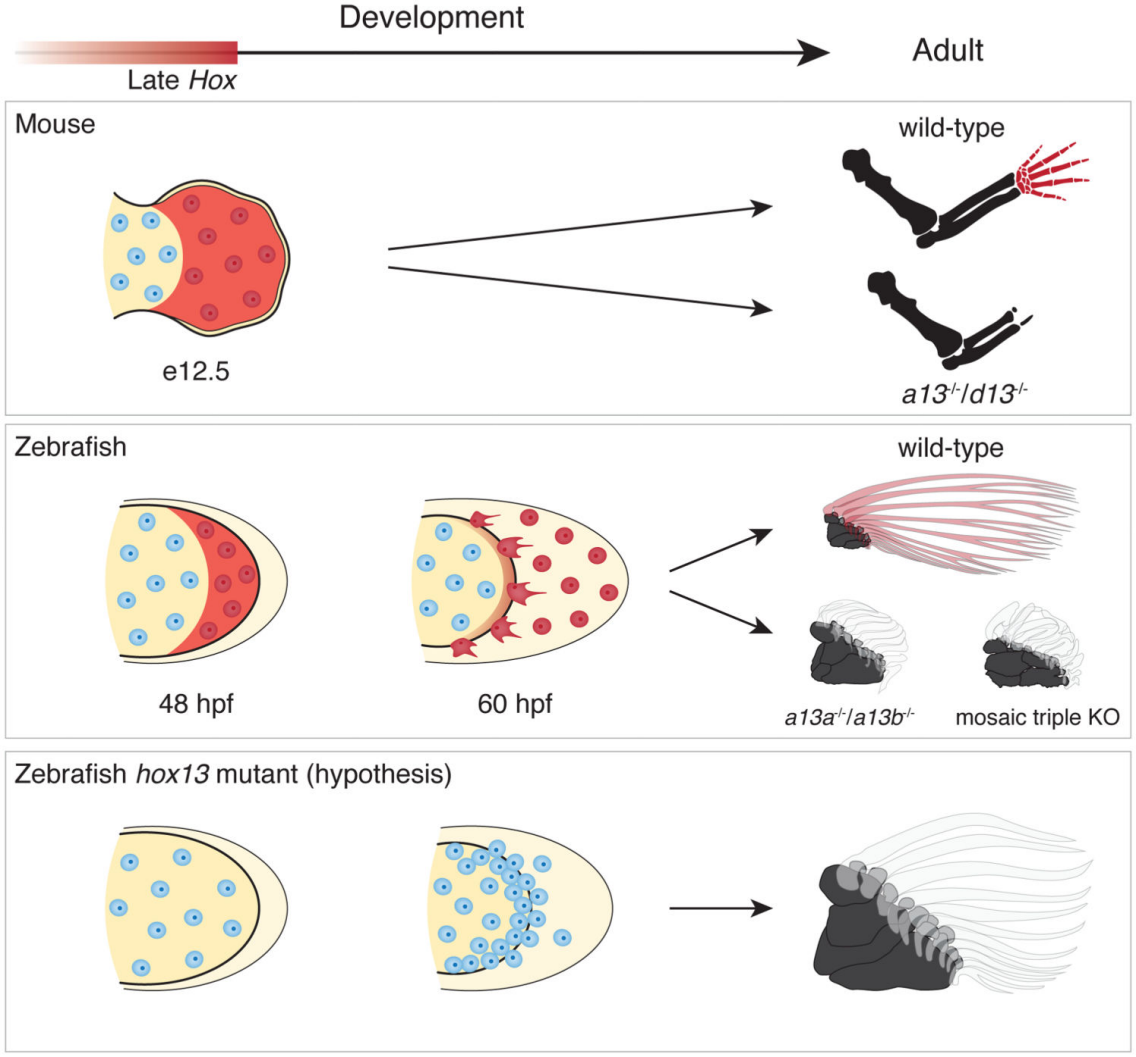
Shared developmental histories in fin rays and digits In mice (top row), late phase *Hox* expression (red shaded) marks the distal cells of the limb bud that result in bones of the autopod (wrists and digits). Double knockout of *Hoxa13* and *Hoxd13* results in the loss of the autopod. In zebrafish wild-type fins (middle row), cells marked by late phase *hox* expression (red shade) end up in the fin fold and within osteoblasts of the dermal rays. *Hoxa13* knockout fish (*hoxa13a*^-/-^, *a13b*^-/-^) and the triple knockout (mosaic for *hoxa13b* and *hoxd13a*) have extremely reduced fin rays with increased distal endochondral radials. Note that distal radials are stacked along the proximodistal axis in the posterior of the fins. The results suggest the hypothesis (bottom row) that the knockout phenotype results from a deficit in migration of mesenchymal cells with more cells left in the distal fin bud (increased number of cells in the endochondral disk of mutants fins, Extended Data Fig. 4) and fewer migrating to the fold, thereby resulting in a larger number of endochondral bones and reduced dermal ones. Red shade: late phase *Hox* expression. Red cells: cells that experienced late phase *hox* expression. Mouse limbs consist of only endochondral bones, but fish fins contain endochondral (black) and dermal (transparent; fin rays) bones.
