# Supplementary material for: Digits and Fin Rays Share Common Developmental Histories
Source: Nature. Author manuscript; Available in PMC 2017 Feb 17. (PMC5161576; doi:10.1038/nature19322)
Supplement: supp_info [file NIHMS804903-supplement-supp_info.pdf]

Wild-type

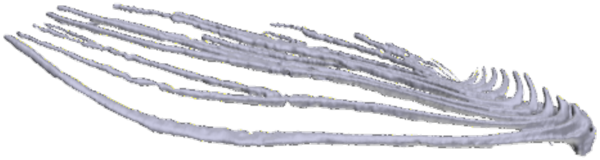

Wild-type radials

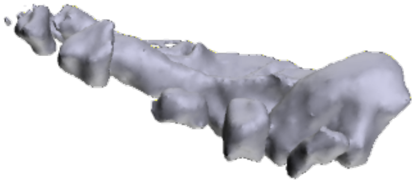

a13a-/- a13b-/-

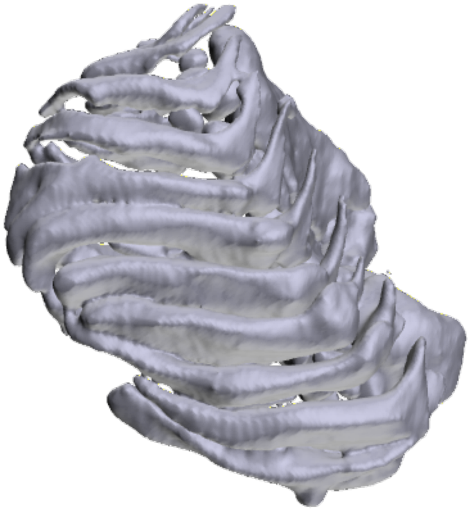

a13a-/- a13b-/- radials

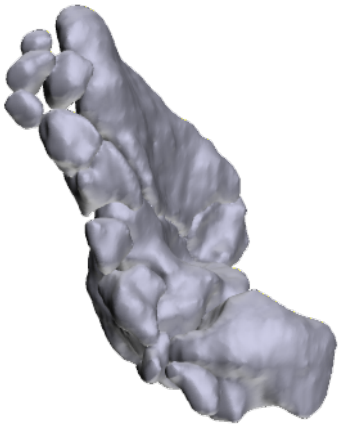

Supplementary Information 3D PDF of wild-type and double mutant fin. Top row: wild-type fin (left) and only endochondral bones (right). Bottom row: hoxa13a -/-, a13b-/- double mutant fin (left) and endochondral bones (right). Compared with wild-type, the fin rays are extremely short in double homozygous fish with the increase of distal radials. Note that distal radials are stacked along proximodistal axis in the mutant fin. After CT segmentation by Amira 3D software, data were output to MeshLab and were converted to 3D PDF.
